# First Complete Genome Sequence of Zika Virus (*Flaviviridae*, *Flavivirus*) from an Autochthonous Transmission in Brazil

**DOI:** 10.1128/genomeA.00032-16

**Published:** 2016-03-03

**Authors:** Mariana Sequetin Cunha, Danillo Lucas Alves Esposito, Iray Maria Rocco, Adriana Yurika Maeda, Fernanda Gisele Silva Vasami, Juliana Silva Nogueira, Renato Pereira de Souza, Akemi Suzuki, Marcelo Addas-Carvalho, Maria de Lourdes Barjas-Castro, Mariângela Ribeiro Resende, Raquel Silveira Bello Stucchi, Ilka de Fátima Santana Ferreira Boin, Gizelda Katz, Rodrigo Nogueira Angerami, Benedito Antonio Lopes da Fonseca

**Affiliations:** a Division of Vector-Borne Diseases, Adolfo Lutz Institute, São Paulo, Brazil; b Department of Internal Medicine, Division of Infectious Diseases, School of Medicine of Ribeirão Preto, University of São Paulo, Ribeirão Preto, São Paulo, Brazil; c Blood Center, State University of Campinas/UNICAMP, Campinas, São Paulo, Brazil; d Section of Hospital Epidemiology, Hospital of Clinics, State University of Campinas/UNICAMP, Campinas, São Paulo, Brazil; e Clinical Medical Department, Faculty of Medical Sciences, State University of Campinas/UNICAMP, Campinas, São Paulo, Brazil; f Infectious Diseases Surveillance Center “Prof. Alexandre Vranjac”/CCD, São Paulo, São Paulo, Brazil

## Abstract

We report here the genome sequence of Zika virus, strain ZikaSPH2015, containing all structural and nonstructural proteins flanked by the 5′ and 3′ untranslated region. It was isolated in São Paulo state, Brazil, in 2015, from a patient who received a blood transfusion from an asymptomatic donor at the time of donation.

## GENOME ANNOUNCEMENT

Zika virus (ZIKV) (family *Flaviviridae*, genus *Flavivirus*) is a single-stranded positive-sense RNA virus that was first identified in 1947 in the Zika Forest, Uganda, and, until recently, was prevalent only in Africa and Asia (1). The virus is primarily transmitted by a mosquito vector, usually from *Aedes* spp., but it is also possible to be transmitted by sexual contact, perinatally, or by blood transfusion (2, 3). Clinical manifestations resulting from infection with this virus are very similar to those caused by other flaviviruses, especially dengue viruses, and include fever, malaise, headache, dizziness, anorexia, retro-orbital pain, and maculopapular skin rash (4). Although most acute infections are asymptomatic, overt symptoms are milder compared to other mosquito-borne virus infections but might be associated to some neurological complications, such as Guillain-Barré syndrome and, more recently, as detected in Brazil, to an impairment of fetal development issues (microcephaly babies) (5, 6). ZIKV was introduced recently in the Americas, as the Ministry of Health of Brazil confirmed autochthonous transmission of ZIKV in the northeastern part of the country in May 2015. Since then, autochthonous transmission of Zika virus has been confirmed in many states of Brazil (7). The ZIKV nucleotide sequence reported here is from a virus isolated from a patient who received a blood transfusion from an otherwise asymptomatic donor. RNA was extracted and cDNA was synthetized prior to sequencing by the Ion Torrent platform (Ion Personal Genome Machine Sequencer, ThermoFisher), and the whole ZIKV genome sequence was obtained by using a 316 chip. The genome assembly was performed using Geneious R8 software, and the reads were assembled using other ZIKV genomes available on GenBank as references; this resulted in a 10,676-bp sequence with an overall G/C content of 51.2%. Per the Phred quality score, 83.5% of the bases were ≥Q20.

This is the first full-length sequence of ZIKV reported in Brazil, and the initial analysis of this Brazilian ZIKV genome shows that, like other flaviviruses, it consists of a linear RNA, which encodes a polyprotein in one unique “long open reading frame” containing all structural protein genes at the 5′ portion of the genome and the nonstructural protein genes at the 3′ portion. The genome organization of flaviviruses, concerning the protein expression order is 5′ C-prM-E-NS1-NS2a-NS2b-NS3-NS4a-NS4b-NS5 3′ (8). The genome sequence of the ZIKV reported here can lead to a better understanding of molecular epidemiology and phylogeny of this strain. Also, it may be of use as a starting point to study genome changes that could possibly explain why microcephaly was detected in Brazil and not in other countries.

### Nucleotide sequence accession number.

The complete genome of Zika virus, ZikaSPH2015 strain, has been deposited in the GenBank under the accession number KU321639.
